# Positive Variables in Adult Patients Who Are at Different Stages of a Naturalistic Psychotherapeutic Treatment

**DOI:** 10.5964/ejop.v14i4.1546

**Published:** 2018-11-30

**Authors:** Vanesa C. Gongora

**Affiliations:** a National Scientific and Technical Research Council (CONICET), Universidad de Palermo, Buenos Aires, Argentina; Department of Psychology, Webster University Geneva, Geneva, Switzerland; Institute of Psychology, University of Wroclaw, Wroclaw, Poland

**Keywords:** positive variables, treatment, patients, adults, therapist

## Abstract

This study aimed twofold: 1) to study some positive variables (three paths to well-being, life satisfaction, overall well-being and meaning of life) in adult patients who are at different stages of a naturalistic cognitive behavioral psychotherapeutic treatment and 2) to analyze their relationship with the progress during treatment, therapeutic alliance and adherence to treatment from the therapist´s perspective. The sample was composed of 85 outpatients who were in psychotherapeutic treatment. Patients completed the Three Pathways to Well-being Scale, Meaning in Life Questionnaire, Satisfaction with Life Scale, Well-being Index and Symptom Checklist-90-Revised. Therapists completed treatment related data and an opinion survey of patient´s progress, adherence to treatment and therapeutic relationship. Findings showed positive variables to be higher at the final stage of psychotherapy, particularly higher satisfaction with life, engagement, well-being, and presence of meaning in life. Higher positive variables were moderately associated with more progress during treatment according to therapist’s perspective; however a low association was found with adherence to treatment and therapeutic relationship. No differences were found in positive variables according the type of prevalent symptoms.

Positive psychology is the scientific study of positive experiences, positive individual traits and the institutions that facilitate their development (Duckworth, Steen, & Seligman, 2005). In the area of clinical psychology, positive psychology aims to broaden the focus on suffering and its direct alleviation to include the development of well-being and optimal functioning (Duckworth et al., 2005). In this area most of research has been focused on positive interventions in student and general population with clinical symptoms, and in some specific clinical groups, particularly in depressive, substance abuse, and posttraumatic stress disorder. These interventions showed to have small to moderate effect sizes to increase well-being and life satisfaction as well as to reduce depressive symptoms (Bolier et al., 2013; Schrank, Brownell, Tylee, & Slade, 2014; Seligman, Rashid, & Parks, 2006; Sin & Lyubomirsky, 2009).

However, less attention had been paid to the study of positive variables in patients who are in psychotherapy as usual treatment. Most of these studies have focused on clinical patients at different stages of treatment taken as a whole group, comparing them with non-clinical groups or with other specific clinical disorder group. Among positive variables most frequently included in studies with patients in treatment are: positive emotions, well-being, satisfaction with life and, more recently, meaning in life.

Numerous studies have highlighted the low level of positive emotions mainly in people with depression (Santos et al., 2013) but also there is some evidence in individuals with anxiety disorders (Watkins & Pereira, 2016) and social phobia (Watson & Naragon-Gainey, 2010). Individuals who received the diagnosis of a mental disorder perceived themselves with lower positive emotions and life satisfaction than those who did not have any disorder and within people who had a mental disorder; those who received a diagnosis of depression had lower levels of well-being (Bergsma, ten Have, Veenhoven, & de Graaf, 2011). Among psychiatric patients, lower levels of life satisfaction were related to depressive symptoms but not to other type of symptoms, being life satisfaction particularly low in this clinical group (Koivumaa-Honkanen et al., 1996). In addition, higher levels of meaning in life were associated to lower symptoms of posttraumatic stress disorder and presence of trauma (Feder et al., 2013; Triplett, Tedeschi, Cann, Calhoun, & Reeve, 2012). Students with clinical depression had lower levels in the pleasant life (predominance of positive emotions), in the engaged life (highly engagement in what one does and experience of flow) and in the meaningful life (using signature strengths and talents to serve something greater than the self) than students without depression and nondepressed patients (Seligman et al., 2006). To sum up, these studies showed positive variables to be low in patients in comparison to non- clinical population and among psychiatric patients those with depressive symptoms seems to have lower positive variables.

In relation to positive variables in patients at different stages of psychotherapeutic treatment, there are only two studies, to our knowledge, that focused in patients at the final stage of treatment. These studies have examined positive variables in patients who were in the final stage of cognitive behavioral treatment for mood and anxiety disorders including panic disorder and agoraphobia. Remitted patients revealed significantly low levels on multiple aspects of psychological well-being; although they had low levels of psychiatric symptoms and could be discharged from treatment, psychological well-being was still low (Fava et al., 2001; Rafanelli et al., 2000).

These findings are in line with two assumptions of positive psychology. First, positive traits and well-being do not necessary improve with treatment as usual and they would require other type of intervention: a positive intervention (Fava, 1999; Seligman, 2002). Second, positive mental health (emotional, psychological and social well-being) and mental illness are two different but related variables. Since they are not inversely related, the absence of a mental disorder does not imply the presence of high levels of well-being (Keyes, 2005). Numerous empirical studies have supported this second assumption (Gilmour, 2014; Lim, Ko, Shin, & Cho, 2013; Petrillo, Capone, Caso, & Keyes, 2015; Yin, He, & Fu, 2013). Nevertheless, an alternative perspective sustained that positive and negative well-being often exists on the same continuum (Wood & Tarrier, 2010). All positive and negative variables have an inverse (e.g. depression vs happiness) and the focus of one extreme of the continuum, either positive or negative, depends on the interest of the clinician or researcher (Johnson & Wood, 2017).

A second interest to study positive variables in patients in treatment is their relationship with progress during therapy and with treatment related process variables. Keyes (2007) sustains that those who have lower levels of well-being tend to have more chronic disorders and less progress in treatment than those with moderate or high levels of well-being. In addition, those individuals with high levels of well-being may have a disorder but this would be rather episodic and with rapid remission (Keyes, 2005, 2007). Research about progress during treatment is very scarce and tends to stand the aforementioned assumption. Low level of emotional well-being predicted lower recovery three years after assessment, particularly for depression (Bergsma, ten Have, Veenhoven, & de Graaf, 2011). Similarly, low levels of positive affectivity predict both slower recovery from depressive episodes and an increased risk of subsequent relapses (Clark, Watson, & Mineka, 1994). In addition, positive variables such as meaning in life were good predictors of abstinence and quality of life in patients who finished treatment for substance abuse (Laudet, Morgen, & White, 2006; McGaffin, Deane, Kelly, & Ciarrochi, 2015). The relationship between positive variables and treatment process related variables has not been examined yet.

The first aim of this research is to study some positive variables (three paths to well-being, life satisfaction, overall well-being and meaning of life) in adult patients who are at different stages of a naturalistic cognitive behavioral psychotherapeutic treatment.

Based on previous findings, it could be expected that lower level on the positive variables would be associated to higher psychiatric symptom and would predominate in the initial phase of treatment in comparison with the final phase. Patients with predominantly depressive symptoms would have lower levels positive variables than patients with other predominant symptoms such as anxiety disorders.

A second aim is to study positive variables in relation to some treatment variables such as progress during treatment, therapeutic alliance and adherence to treatment from the therapist´s perspective.

Therapist´s report has shown to be a very valuable source of information of treatment process providing a different and complementary contribution to study therapeutic process and outcome. Therapists reports are also relevant to understand common or non-specific therapy factors such as therapeutic alliance and patient commitment and these reports were found to be better predictors of outcome than patients reports (Bachelor, 2013; Clemence, Hilsenroth, Ackerman, Strassle, & Handler, 2005; Laska, Gurman, & Wampold, 2014).

It is expected that patients with higher positive variables would be perceived with greater therapeutic progress by therapist. In addition, since positive variables such as well-being, meaning in life, positive emotions and engagement have been associated to better and more intimate relationships (Coffey, Wray-Lake, Mashek, & Branand, 2016; Keyes, 2005), it could be expected that patients with higher levels of these variables would be perceived by therapist with better therapeutic alliance and higher adherence to treatment

## Method

### Participants

The sample was composed of 85 outpatients (25 men and 60 women) who were in psychotherapeutic treatment in five mental health services (private and public) in the city of Buenos Aires. Patients mean age was 40.83 years (*SD* = 16.0; range = 18-75 years). A 53.1% were middle-aged adults (31-60 years), a 34.9% were young adults (18-30 years) and only a 12% were seniors (+61 years). Exclusion criteria were: being inpatient, receiving a diagnosis of schizophrenia and other psychotic states, dementia and cognitive disorders, or bipolar disorders as well as those whose state of crisis prevented them from completing the questionnaires. With regard to the type of pathology, 58.8% had an anxiety disorder, 21.2% a depressive disorder, 17.6% a comorbid disorder with anxiety and depressive symptoms and 2.4% some other disorder.

Therapist who treated study patients: 11 men and 7 women. They were licensed psychologist, who had at least 5 years of clinical experience on cognitive-behavioral treatment. They had no training in positive interventions.

Treatment was cognitive-behavioral oriented. Therapists did not have any particular training or instruction about the treatment they provided during this research, psychotherapy was “as usual”. They provided cognitive-behavioral interventions they deemed appropriate for their patients without following a particular treatment protocol. In addition to the psychotherapy, 62.4% of patients also assisted to psychiatric control and received medication (mainly antidepressant and anxiolytic medication). The average duration of treatment was 5.15 months (*SD* = 4.94). Duration of treatment depended on patient progress. Criteria to consider the stages of treatment were provided to therapist. The initial stage corresponded to the first treatment sessions in which the patient's symptoms were assessed, the diagnosis was formulated, the therapeutic relationship with the patient begun and the aims and strategies for treatment were established. The intermediate stage was the part where treatment was properly developed. The therapist employed therapeutic techniques and strategies on the basis of treatment aims. It is usually the longer and main part of treatment. The final stage was considered when the therapeutic aims were practically fulfilled, symptoms have remitted and work was being done on the discharge of treatment. It was assumed that the duration of the stages depended on the characteristics of the patients; for example, some patients would need more assessment time while others less. The mean duration of treatment was 1.09 months (*SD* = .9) for patients at initial stage, 5.75 months (*SD* = 4.44) for patients at intermediate stage and 9.87 months (*SD* = 5.5) for those at final stage. Concerning the stage of treatment, 22 patients were at initial stage, 46 at an intermediate stage and 17 at a final stage.

### Instruments

#### Three Pathways to Well-Being Scale (TPWB)

The TPWB assesses well-being according to the three pathway model proposed by Seligman (2002). It contains 23 statements rated on a 5-point Likert scale that ranges from 1 (very different from me) to 5 (very similar to me). The TPWB is divided into three subscales, each of which corresponds to one of the three pathways to well-being: the pleasant life (referring to the maximization of positive emotions to achieve pleasure); the engaged life (concerning having goals and using one’s strengths to achieve them), and the meaningful life (regarding the use of personal strengths to serve the social environment beyond oneself). The scale was developed for and validated in Argentinean adults (Castro Solano, 2011). The TPWB showed adequate evidence of validity and reliability in adult and adolescent population in Argentina (Castro Solano, 2011; Góngora & Castro Solano, 2015). Exploratory and confirmatory factor analyses verified the three-factor structure of the test. The internal consistencies for the three subscales were α ≥ .70 in adult and adolescent validation samples. (Castro Solano, 2011; Góngora & Castro Solano, 2015). In the current sample internal consistency was α = .81 for the pleasant life, α = .83 for the engaged life, and α = .78 for the meaningful life.

#### Meaning in Life Questionnaire (MLQ)

The MLQ is a 10-item scale that assesses the extent to which respondents feel that their lives are meaningful (Steger, Frazier, Oishi, & Kaler, 2006). The MLQ is composed of two independent subscales: Search for Meaning and Presence of Meaning. Each dimension of meaning is measured via 5 items that are rated from 1 (absolutely untrue) to 7 (absolutely true). The two-factor structure of the MLQ has been replicated using confirmatory factor analysis (CFA) in multiple samples across different cultures, including in Argentine adults (Góngora & Castro Solano, 2011; Steger et al., 2006). Convergent validity was established in adult and adolescent Argentine samples. Associations were moderate to high with the Presence of Meaning and low to moderate with the Search for Meaning. Internal consistencies for the subscales were α ≥ .80 (Góngora & Castro Solano, 2011). Internal consistencies in the current sample for the subscales were α = .87 for Presence of Meaning and α = .89 for Search for Meaning.

#### Satisfaction With Life Scale (SWLS)

The SWLS is a 5-item scale that assesses overall life satisfaction (Diener, Emmons, Larsen, & Griffin, 1985). Respondents rate each item from 1 (strongly agree) to 7 (strongly disagree). The SWLS is among the most widely used measures of well-being, and various international empirical studies, including in Argentina, have demonstrated its validity and reliability (Castro Solano, 1999; Diener et al., 1985; Pavot, Diener, Colvin, & Sandvik, 1991). In Argentinean adult and adolescent samples, the scale has confirmed the unidimensional factorial structure, and the convergent validity with psychological well-being, and job satisfaction. The reliability reported in the validation studies was α ≥ .72 (Castro Solano & Casullo, 2001; Dimitrova & Dominguez Espinosa, 2015; Moyano, Martínez Tais, & Muñoz, 2013). In this sample, the internal consistency as measured by Cronbach's alpha was α = .87.

#### Well-Being Index

This is a short scale of 5 items in a 10 point Likert scale that assesses jointly the level of hedonic and eudaemonic well-being. Each of the items refers to an overall perception of happiness, satisfaction with life and the three pathways of well-being: positive emotions, engagement and meaning in life. Studies have demonstrated, through exploratory and confirmatory factor analysis, the adequacy of a single factor structure in Argentine samples of adolescents and adults. The reported reliability was .80 for the sample of adults and .84 for adolescents. (Góngora & Castro Solano, 2015). In the current sample the internal consistency was α = .89.

#### Symptom Checklist-90-Revised (SCL-90-R)

This is a 90-item checklist (Derogatis, 1977) used to measure nine sets of psychological symptoms: Somatization, Obsessive-Compulsive, Interpersonal Sensitivity, Depression, Anxiety, Hostility, Phobic Anxiety, Paranoid Ideation, and Psychoticism. The SCL-90-R also contains a Global Severity Index (GSI), which is used to estimate the “general psychiatric status” of a patient. The SCL-90-R uses a 5-point scale (1 = “no problem” to 5 = “very serious”) to measure the extent to which they have experienced the listed symptoms in the last 7 days. Studies with the Argentinean adaptation of the checklist have shown adequate internal consistency for all scales (all αs > .72) and have replicated the original factorial structure in general and psychiatric populations (Casullo, 2004; Sanchez & Ledesma, 2009). Internal consistency for the total scale in this sample was α = .97.

#### Treatment Related Data Sheet

Therapist were asked to complete a data sheet about the initial symptoms of patients, duration of treatment, treatment stage (initial / intermediate / final), primary diagnosis, and previous psychiatric/psychological treatment.

#### Opinion Survey of Patient´s Progress, Adherence to Treatment and Therapeutic Relationship

 Therapists were asked to complete a total of 5 items opinion survey about their patients. Two items were about: the progress they considered patients have from the start of treatment (progress in relation to the initial complaints and progress in relation to the main symptoms). They were assessed in a 5-option Likert scale (worst, equal, a bit better, much better, excellent progress). Two other items referred to the adherence to treatment assessed through compliance schedules and attendance to meetings, and about the compliance of suggestions or indications of the therapist (bad, fair, good, very good, excellent). A last item asked the therapist´s opinion about the therapeutic relationship (bad, fair, good, very good, excellent).

### Procedure

Therapists were requested to select patients who were able to participate in the study according to the inclusion and exclusion criteria mentioned in the sample section. Patients were informed of the research aims and completed an informed consent. The administration of the tests was performed individually during a single session of 45 minutes in the presence of a member of the research team. Therapist completed the patient´s data sheet and the opinion survey about patient´s progress in a single session of 5-10 minutes.

### Type of Design

A cross-sectional design was used in this study. Positive variables were assessed in patients who were at different stages of a naturalistic cognitive-behavioral oriented treatment. Treatment was “as usual” and no manipulation of any variable was made.

### Statistical Analyses

Patients were divided into groups of treatment stage according to the information provided by therapist. In the Treatment Related Data Sheet therapists informed the stage of treatment patients were at the time of study assessment (initial/intermediate/final). Criteria to consider the stages of treatment have been detailed in the Sample section.

The SPSS 18.0 program was used in the data analyses. A Multivariate Analysis of Variance (MANOVA) was chosen to compare positive variables among patients at different stages of treatment due to this type of analysis provide a general main effect for all dependent variables. Tukey post -hoc univariate analyses were also performed. The same statistical procedure was carried out in separate analyses using previous psychiatric treatment (yes/no), previous psychological treatment (yes/no) and the type of symptoms (anxiety, depressive, both anxiety and depressive and other) as factors. Pearson correlations were calculated to examine the relationship between positive variables and duration of treatment and between positive variables and duration of illness.

Given that there were several positive measures, Principal Axis Factor Analysis with Oblimin rotation was performed to reduce the number of variables. Kaiser’s criterion of retaining factors with eigenvalues greater than 1 was used. Factor loadings of .40 or greater were considered for item retention. A positive factor was obtained through this procedure. The same statistical procedure was used with the therapist´s opinion of progress variables: progress in relation to the initial complaints and progress in relation to the main symptoms. A progress factor was also obtained in this second analysis. Finally, Pearson correlations were calculated between the positive factor, the progress factor, the SCL-90 –GSI score, and the treatment process related variables according to therapist´s opinion (compliance of therapeutic indications, attendance to treatment and therapeutic relationship).

## Results

### Positive Variables at Different Stages of Treatment

Positive variables in this study were: satisfaction with life, presence and search of meaning in life, overall well-being (hedonic and eudaemonic), and three pathways to well-being: pleasure, engagement and meaning. A Multiple Analysis of Variance was performed in order to compare positive variables among stages of treatment: initial, intermediate, final. An almost significant main effect of positive variables on the stages of treatment was found (Wilks λ = .74, *F* = 1.71, *p* = .04, η^2^ = .14). Results are presented in Table 1.

**Table 1:** Comparison of Means of Positive Variables Between Patients at Different Stages of Treatment

Variables			Stage of Treatment							
	Total		Initial		Intermediate		Final		*F*	η^2^
	*M*	*SD*	*M*	*SD*	*M*	*SD*	*M*	*SD*		
Satisfaction with Life	20.64	7.63	19.90_a,b_	6.54	19.29_a_	8.04	25.05_b_	6.41	3.88*	.09
Pleasant life	29.63	6.17	30.57	5.97	28.61	6.34	31.11	5.79	1.34	.03
Engaged life	25.60	5.68	25.62^a,b^	5.45	24.13_a_	5.61	29.41_b_	4.55	5.91**	.13
Meaningful life	26.43	6.49	26.28	6.50	25.65	6.29	28.64	6.88	1.31	.03
Overall well-being	32.23	10.25	29.28_a_	11.90	31.61_a,b_	9.83	37.47_b_	7.32	3.35*	.08
Presence of meaning	22.82	7.87	19.90_a_	8.04	22.77_a,b_	7.85	26.58_b_	6.41	3.60*	.08
Search for meaning	21.11	8.78	21.42	8.24	22.09	8.73	18.17	9.40	1.24	.03

*Note.* Means sharing a common subscript are not statistically different at α = .05 according to the Tukey HSD procedure.

*p < .05. **p < .01.

Univariate post-hoc analysis indicated significant differences with moderate to large effect sizes in: satisfaction with life (*F* = 3.88; *p* = .02; η^2^ = .09), engaged life (*F* = 5.91; *p* = .004; η^2^ = .13), overall well-being (*F* = 3.35; *p* = .04; η^2^ = .08), and presence of meaning in life (*F* = 3.60; *p* = .03; η^2^ = .08). In the aforementioned variables means were higher in the final stage in comparison with the intermediate and initial stages of treatment.

### Positive Variables, Chronicity of Illness and Type of Symptoms

A similar procedure was carried out in order to compare positive variables between patients who had a previous psychiatric treatment (*n* = 30) and those who had not (*n* = 55). No significant differences were found between the two groups (Wilks λ = .94, *F* = .59, *p* = .76). Similarly, positive variables did not differ between patients who had a previous psychological treatment (*n* = 63) and those who had not (*n* = 22) (Wilks λ = .93, *F* = .75, *p* = .62). No positive variables were correlated to duration of illness but the presence of meaning in life was moderately associated to duration of treatment *r* = .29, *p* = .007.

No significant differences were found among positive variables and the type of symptoms (anxiety, depressive, both anxiety and depressive and other) patients had at intake (Wilks λ = .74, *F* = 1.08, *p* = .34).

### Positive Variables, Progress During Treatment and Treatment Process Related Variables According to Therapist Opinion

In order to analyze the relationship between positive variables and the therapist´s opinion about progress during treatment and to reduce the large number of positive variables, a principal axis exploratory factor analysis (EFA) with Oblimin rotation was performed introducing all positive measures. First, two factors were obtained. The first one included most of positive variables, but the variable pleasant life loaded simultaneously in both factors and search for meaning in the second factor. A second EFA was conducted excluding the pleasant life. In this analysis, the first factor comprised all positive variables with the exception of search for meaning which loaded in the second factor. Since the second factor included only one variable and the all the other positive variables loaded in the first factor, a new EFA was performed excluding the search for meaning. The EFA showed an adequate relationship between the variables and number of participants (KMO = .81, χ^2^ = 191.75, *df* = 10, *p* < .001). The EFA resulted in one factor which explained 64.59% of the variance. Items loading were all above .69. This positive factor contained the variables: satisfaction with life, presence of meaning in life, overall well-being (hedonic and eudaemonic), engaged life and meaningful life.

In order to reduce the therapist´s opinion of progress, a new exploratory factor analysis was performed with the two variables: progress in relation to the initial complaints and progress in relation to the main symptoms. This EFA obtained one factors that explained 91.5% of total variance (KMO = .58, χ^2^ = 124.91, *df* = 6, *p* < .001). Items loaded above .95 in the factor.

**Figure 1:**
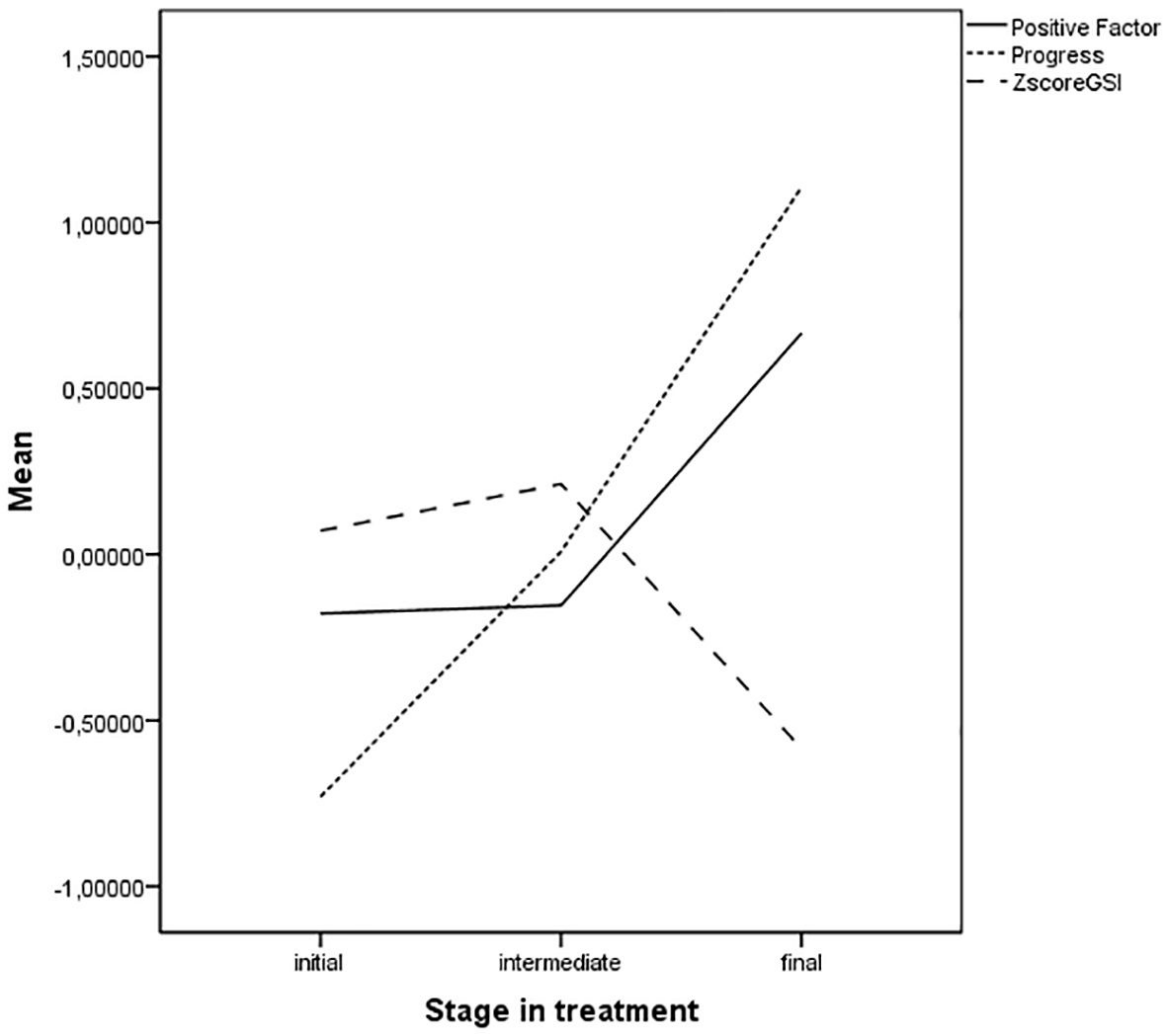
Means scores of positive factor, progress factor and SCL-90 –GSI by stage of treatment.

Means of the positive factor, progress factor and psychiatric symptoms (measured through the SCL-90 –GSI score) by stage of treatment are presented in Figure 1. GSI scores were transformed into Z-scores in order to present them in the same type of scores as the factor variables (mean = 0).

The progress factor clearly increases from initial stage to final stage of therapy ranging from very low score to very high scores. In other words, those patients who were at initial stage were perceived by therapist with low progress, those who were in an intermediate stage were perceived with a medium progress and those in the final stage with a high progress during treatment. Positive variables and psychiatric symptoms appear to be stable, quite parallel and near mean score in patients who were at initial and intermediate stage of treatment, although GSI score have higher means at these stages than positive variables. Nevertheless, in the final stage of treatment they clearly distinct from each other, being positive variables high and psychiatric symptoms low.

Bivariate correlations were calculated between the positive variables factor, the progress factor, the SCL-90 –GSI score, and the treatment process related variables according to therapist´s opinion. Results are presented in Table 2.

**Table 2:** Correlations Between the Positive Factor, Progress Factor, SCL-90 GSI, and Treatment Process Related Variables

Variables	1	2	3	4	5	6
1. Positive variables	–					
2. Progress during treatment	.28*	–				
3. GSI	-.56**	-.18	–			
4. Compliance to schedules and attendance to meetings	.07	.31*	-.30*	–		
5. Adherence to suggestions and therapeutic indications	.19	.61**	-.23	.66**	–	
6. Therapeutic Relationship	.13	.40**	-.18	.61**	.55**	–

*p < .05. **p < .01.

Positive variables had a moderate correlation (*r* = .28) with the perception the therapist reported about the progress patient had during treatment. In addition, positive variables were strongly and negatively correlated with the level of general psychiatric symptomatology (*r* = -.56). Small correlation were found between positive variables and the therapist´s opinion about compliance of therapeutic suggestions or indications (*r* = .19), about compliance schedules and attendance to meetings (*r* = .07) and about therapeutic relationship (*r* = .13).

Concerning the relationship between the general psychiatric symptomatology and the therapist´s perception of progress during treatment, correlations were negative and low (*r* = -.18), as well as with therapist´s opinion about compliance schedules and attendance to meetings (*r* = -.23) and about therapeutic relationship (*r* = -.18). Nevertheless, there was a moderate correlation with therapist´s opinion about compliance of therapeutic suggestions or indications (*r* = -.30).

Therapist´s perception of progress during treatment were more significantly associated with the same therapist´s report of compliance schedules and attendance to meetings (*r* = .31), quality of therapeutic relationship (*r* = .40) (moderate effect size) and strongly to compliance of therapeutic suggestions or indications (*r* = .61). Among the three therapy process related variables, correlations were very strong (*r* = .55 to *r* = .66).

## Discussion

This study showed that positive variables were higher in patients who were at the final stage of a naturalistic cognitive behavioral treatment; in particular, they had higher satisfaction with life, engagement, well-being, and presence of meaning in life. These positive variables tended to be relatively stable and low during the initial and intermediate stage of treatment, but they are significantly higher in the group attending the final stage.

Although previous studies have found that psychological well-being to be low in remitted patients with mood and anxiety disorders (Fava et al., 2001; Rafanelli et al., 2000), this study shows that those patients who are at the final stage have low psychiatric symptoms and high positive variables. This is a very interesting finding considering that it is a naturalistic treatment with no positive interventions included among techniques. It also contradicts one of the assumptions of positive psychology that posits that positive variables do not necessary increase with a treatment as usual as cognitive behavioral treatment (Seligman et al., 2006). Nevertheless, the cross-sectional nature of the study does not allowed to know whether those who had higher levels of positive variables at initial stage were those who reached the final stage of treatment or whether positive variables increased during treatment. If positive characteristics increased during therapy some possible explanations can be argued. On the one hand, Wood and Tarrier (2010) have sustained that positive and negative characteristics often exists on the same continuum (Wood & Tarrier, 2010), for instance in one negative extreme could be depression and in the positive one happiness. Johnson and Wood (2017) add that all negative variables have a positive inverse variable. In this study a very strong correlation between positive variables and psychiatric symptoms was found, thus, it may be possible that the decrease of psychopathology at the final stage of therapy derived to an increase of positive traits. On the other hand, Duckworth, Steen, and Seligman (2005) suggested that many therapists include some strategies with their patients that, in fact, are very compatible with positive psychology. These strategies may refer to inspiring hope, building strengths such as courage, interpersonal skill, optimism, perseverance, pleasure capacity, personal responsibility, or purpose (Duckworth et al., 2005). Thus, some non-specific interventions may contribute to increase the positive traits in patients, although it is not a positive psychotherapy. Since there are almost no study that examine positive variables in treatment as usual, there would be necessary longitudinal studies to explore the consistency of these findings and clarify if there is only a subgroup of patients with certain intake characteristics that complete their treatment with high positive characteristics or independently of intake reports all patients who are in the final stage have these positive traits due to the therapeutic process and the reduction of symptoms.

Higher meaning in life has been associated with better outcome and it was a good predictor of success in addiction treatment (Laudet et al., 2006; McGaffin et al., 2015). In this study we found higher meaning in life in a naturalistic cognitive-behavioral treatment mainly for depression or anxiety disorders. In addition, a higher meaning in life was associated to a longer duration of treatment. Longer treatments have been found to be related to better therapeutic outcomes including better functioning in a number of specific and general life areas (Clemence et al., 2005). This study adds that longer treatments provide higher presence of meaning in patient´s life.

Contrary to expectations, no differences were found in any positive variable according to the type of predominant symptomatology. Many studies have found lower positive variables in patients with depression (Bergsma et al., 2011; Koivumaa-Honkanen et al., 1996; Seligman et al., 2006). There were almost no study with patients with anxiety disorders, but this study showed that anxiety, and depressive patients have similar levels of positive variables. There is strong evidence indicating that similar etiological and maintenance processes underlie depressive and anxious psychopathology (Newby, McKinnon, Kuyken, Gilbody, & Dalgleish, 2015). For instance, anxiety and depressive disorders share many similar genetic, familial, and environmental risk factors (Kendler, 1996). These disorders also share similar cognitive-affective, interpersonal, and behavioral maintaining factors (Harvey, 2004). These similarities seem also to extent to positive characteristics. These etiological and maintenance processes may also lie beneath the development of positive characteristics.

Contrary to expectations, lower positive variables were not associated to chronicity of illness measured by previous psychological or psychiatric treatment and duration of illness. To interpret this finding it should be taken into account the high percentage in the sample of patients with previous psychological (74.11%) and psychiatric treatment (35%). This high percentage could be related to the relatively easy access to psychological and psychiatric treatment in public hospitals (even free of cost) or in the private sector in Buenos Aires and also to a culture very disposed to consult psychologists for personal crisis as well as for important symptoms. In a large and stratified study, Escalante and Leiderman (2008) found 78.1% of adult population of Buenos Aires assisted at least once in life to psychotherapy and 15.6% were assisting at the moment of study (Escalante & Leiderman, 2008).

Positive variables were moderately related to the therapist´s perception of progress during treatment. Although moderate, this association was stronger than the perception of progress and psychiatric symptoms, which was low. Thus, in relation to the therapist´s evaluation of progress during treatment: positive variables were more relevant and stronger associated to higher progress than low psychiatric symptoms.

Nevertheless, therapist´s perceived progress was stronger associated to other therapist related variables particularly compliance of suggestions or therapeutic indications, which could be expected. A patient that follows suggestions and therapeutic indications it is logical that is perceived as progressing in treatment, at least from therapist perspective. These findings are very much alike the strong previous evidence on variables related to positive outcome and treatment process (Bachelor, 2013; Clemence et al., 2005). This consistency also shows that, although standardized instruments for therapist´s report of progress, therapy process or therapeutic alliance were not used in this study, results are similar to previous ones.

It should be noted that it is expected a lower association in informant reports in comparison with self-reports (Meyer et al., 2001). Meyer et al. (2001) found that correlations between patient and clinician were low to moderate ranging between *r* = .14 to *r* = .34. That would be one of the reasons why all therapist´s reported variables have a strong correlation among them. This is similar with previous studies with reports of outcome and therapeutic alliance comparing patient vs therapist. All therapists’ variables have higher correlation among them (Bachelor, 2013; Clemence et al., 2005). Considering the aforementioned reports of association between patient and clinician, the correlation between perceived progress and positive variables become more important.

Contrary to expectations, positive variables were not related to any treatment process related variable according to therapist´s report. In the same way, the severity of psychiatric symptoms was neither related to therapeutic relationship or to attendance to treatment, but was moderately related to compliance of therapeutic indications. Previous studies also found that therapist´s rating on therapeutic alliance and treatment collaboration were better predicted by therapist´s ratings such as active engagement or positive valuation rather than to client´s symptomatology or interpersonal relationships (Bachelor, Laverdière, Gamache, & Bordeleau, 2007).

Some limitations should be mentioned. First, the correlational design of the study does not allowed to make causal inferences and to know how patients enter and changed during treatment. It would be important to advance in a longitudinal study in order to stablish the process of change of positive and negative variables. Second, the limited number of patients, although it is not a very small sample number a bigger group, particularly of patients at initial and final stages of treatment would have been desirable. Third, a naturalized study was used. This type of study has the advantage of showing data of patients in more realistic treatment setting (Leichsenring, 2004). The purpose of this study was to examine some positive variables in a treatment as usual for that reason a naturalistic study was chosen. Nevertheless, it is important to consider that as the type of treatment was not controlled for, divergences about implementation of treatment among therapist may affect results. Finally, concerning treatment process related variables, not standardized instruments for progress, therapeutic relation and adherence to treatment were used in this study. Possibly a standardized instrument would have been more valid and reliable; however, results are very consistent with previous researches in treatment process which give us some confidence about the adequacy of instruments. Future studies should deepen the study of therapeutic process and positive variables including also patient’s perspective.

### Conclusions

In sum, this study was the first to compare positive variables among different stages of a naturalistic psychotherapeutic treatment, and we also compared them among different types of prevalent symptoms, including patients with anxiety disorders, group that was very scarce in researches with positive variables. This study also provided a first approach to the relationship between positive variables and the therapist perception of progress in therapy and some treatment process related variables. The study of positive functioning in patients will enrich clinical psychology to become a more integrative discipline. In this way, the aim of therapy will not only be relieving the negative but also helping individuals to build a flourishing life.
